# Optimising Resident Doctor Induction in a Mental Health Trust: A Mixed-Methods Quality Improvement Review

**DOI:** 10.1192/bjo.2026.11259

**Published:** 2026-06-30

**Authors:** Esther Anekwu, Arun Viswanath, Esraa Abdelmajeed, Grace Threlfall

**Affiliations:** 1 Sheffield Health Partnership University NHS Foundation Trust, Sheffield, United Kingdom; 2 South West Yorkshire Partnership NHS Foundation Trust, Barnsley, United Kingdom

## Abstract

**Aims:**

A clear and well-timed induction is important in helping resident doctors work safely and confidently in mental health services. Psychiatry presents specific challenges, including the use of legal frameworks, managing risk, and working within multidisciplinary teams. Feedback from doctors starting in the Trust suggested that the induction programme was variable and that some felt unprepared when beginning clinical work.

This Quality Improvement project aimed to review the current induction programme, identify gaps that affect clinical readiness and patient safety, and support improvements in how the induction is structured and delivered.

**Methods:**

A mixed-methods survey was sent to resident doctors who had completed the Trust induction programme within the previous 12 months. The survey included rating-scale questions on coverage, usefulness, and preparedness, alongside open-ended questions about individual experiences. Quantitative responses were summarised using simple descriptive measures, and free-text responses were reviewed to identify common themes. The project followed a Plan–Do–Study–Act approach. As this work was carried out as a Quality Improvement project, formal ethical approval was not required.

**Results:**

Eight out of ten resident doctors across different training grades responded. Mostfelt that the induction helped them understand their role within psychiatry, but fewer felt prepared for on-call duties and seclusion reviews. Practical sessions, such as IT access, prescribing systems training, Mental Health Act teaching, and breakaway training, were viewed positively, especially when delivered early and in person. Several key areas, including discharge summaries, ward rounds, and educational meetings, were reported as not covered for some doctors. Common concerns included induction sessions being spread over several weeks, limited protected time, and starting clinical duties before completing essential systems training. These factors reduced attendance and confidence. Most respondents felt that online or recorded learning materials would support learning alongside face-to-face teaching.

**Conclusion:**

The induction programme contains important psychiatry-specific content but is less effective due to delays, poor sequencing, and lack of protected time. A shorter, more focused induction period, completion of essential training before clinical duties begin, and a blended approach to teaching may improve preparedness and support safer patient care. These findings will inform a revised induction programme and a further PDSA cycle.

